# Modified Senning Procedure for Treatment of Transposition of the Great Arteries with Crisscross Heart

**DOI:** 10.21470/1678-9741-2023-0244

**Published:** 2024-07-15

**Authors:** Ana Carolina Pereira de Godoy, Marilia Maroneze Brun, Fabiana Nakamura Avona, Carlos Henrique De Marchi, Ulisses Alexandre Croti

**Affiliations:** 1 Department of Pediatrics and Pediatric Surgery, Pediatric Cardiology and Cardiovascular Surgery at CardioPedBrasil® – Hospital da Criança e Maternidade de São José do Rio Preto, São Paulo, Brazil.

**Keywords:** Arterial Switch Operation, Cardiopulmonary Bypass, Delayed Diagnosis, Pulmonary Artery, Crisscross Heart, Transposition of Great Vessels, Heart Ventricles

## Abstract

**Clinical data:**

A nine-month-old female infant diagnosed with transposition of the great arteries with symptoms of heart failure associated with cyanosis and difficulty in gaining weight was referred to our center with late diagnosis (at nine months of age).

**Chest radiography:**

Cardiomegaly; attenuated peripheral vascular markings.

Electrocardiography: Sinus rhythm with biventricular overload and aberrantly conducted supraventricular extra systoles.

**Echocardiography:**

Wide atrial septal defect, ventricular axis torsion with concordant atrioventricular connection and discordant ventriculoarterial connection.

**Computed tomography angiography:**

Concordant atrioventricular connection, right ventricle positioned superiorly and left ventricle positioned inferiorly; discordant ventriculoarterial connection with right ventricle connected to the aorta and left ventricle connected to pulmonary artery.

**Diagnosis:**

Crisscross heart is a rare congenital heart defect, accounting for 0.1% of congenital heart diseases. It consists of the 90º rotation of ventricles’ axis in relation to their normal position; therefore, ventricles are positioned in the superior-inferior direction rather than anterior-posterior. Most cases have associated cardiac anomalies, and in this case, it is associated with transposition of the great arteries. The complexity and rarity of its occurrence make diagnosis and surgical treatment challenging.

**Operation:**

Modified Senning procedure using the pericardial sac in the construction of a tunnel from pulmonary veins to the right atrium. Cardiopulmonary bypass time of 147 minutes with nine minutes of total circulatory arrest.

## CASE PRESENTATION

A female infant born in Dois Riachos (state of Alagoas, Brazil), preterm at 32 weeks, with no diagnosis of congenital heart disease at birth, presented with difficulty in gaining weight (failure to thrive) and progressive cyanosis at five months of age. An echocardiogram diagnosed transposition of the great arteries (TGA). The infant was referred to our center as an outpatient at nine months of age without use of any medications.

Physical examination revealed good general condition and hemodynamic stability. Patient was eupneic with a respiratory rate of 31 breaths per minute. Cyanosis, oxygen saturation of 75%, finger clubbing, and grade 2+/6+ murmur at middle left sternal border were also noted.

## TECHNICAL DESCRIPTION

### Chest Radiography

**Fig. 1:**
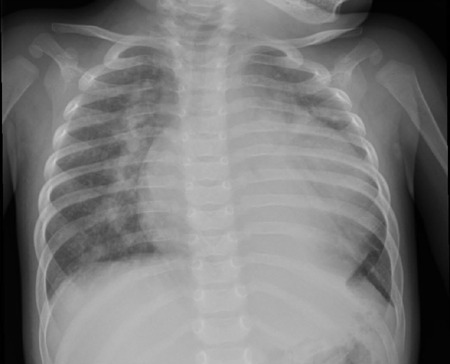
Chest X-ray demonstrating significant cardiomegaly and attenuated peripheral vascular markings.

*Situs solitus* in levocardia and cardiothoracic ratio of 0.68 (cardiomegaly). Attenuated peripheral vascular markings (Figure 1)^[1]^

### Electrocardiography

**Fig. 2:**
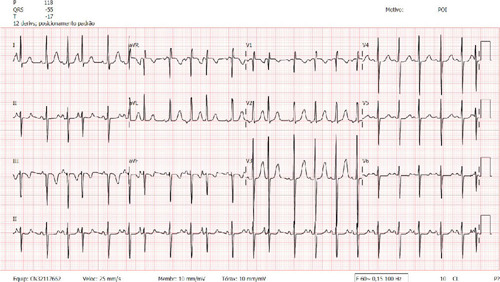
Sinus rhythm, heart rate of 136 bpm, suggestive signs of biventricular overload; anterosuperior divisional block.

Sinus rhythm, heart rate of 136 bpm, suggestive signs of biventricular overload; anterosuperior divisional block (Figure 2)^[1]^.

### Echocardiography

**Fig. 3:**
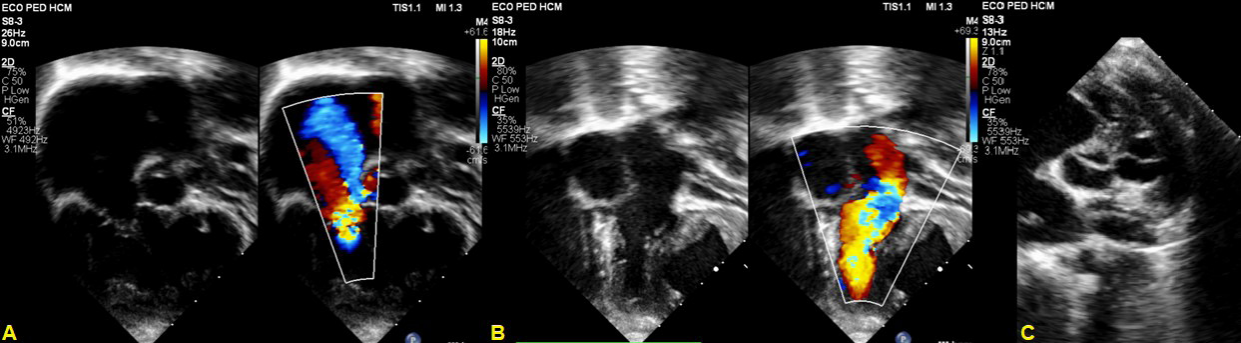
Preoperative echocardiogram. A) Apical image: right ventricle with axis rotation and tricuspid valve insufficiency. B) Apical image: left ventricle crossing the inlet of the right ventricle. C) High short-axis parasternal view: ventriculoatrial valves side by side.

*Situs solitus* in levocardia, usual venoatrial connection. Concordant atrioventricular connection with twisted cardiac axis. Discordant atrioventricular connection, with pulmonary artery on the right side and aorta on the left side. Presence of interventricular septum bulged into the left ventricle (type III) and atrial septal defect (ASD) measuring approximately 12 mm with bidirectional flow. These findings suggest higher pressures on the right side (Figure 3).^[1]^

Significant tricuspid valve insufficiency with right ventricular systolic pressure of 101 mmHg.

Mild dilatation of left pulmonary artery branch (+2,31 z). Other portions of pulmonary artery on usual size. Biventricular systolic function was normal.

### Computed Tomography Angiography

Wide ASD. Right atrium communicating with right ventricle, located superiorly due to cardiac axis rotation. Left atrium communicating with left ventricle, located inferiorly. Right ventricle with prominent trabeculation, connected to the aorta. Left ventricle connected to the pulmonary artery. This description is compatible with crisscross heart (CCH) and TGA.

**Fig. 4:**
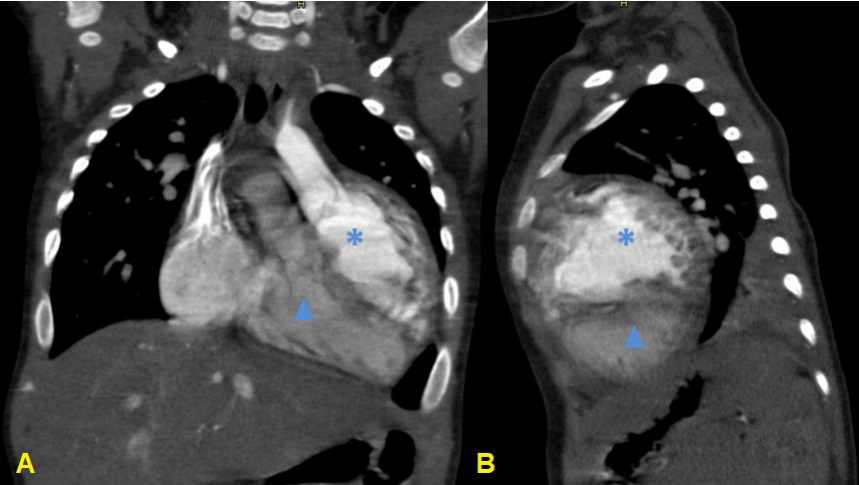
Computed tomography angiography: coronal (A) and sagittal (B) sections showing right ventricle in superior position and left ventricle (solid arrow) in inferior position.

Left pulmonary artery dilatation (+2,58 z). Left main bronchus with extrinsic compression by the left pulmonary branch anteriorly, and posteriorly by the descending thoracic aorta and vertebral column (Figure 4).

## COMMENT

### Diagnosis

CCH is a rare congenital heart defect, first described by Lev and Rowlatt^[1]^ in 1961 and named by Anderson^[2]^ in 1974. It represents 0.1% of congenital heart defects, not exceeding eight per 1.000.000 live births^[3]^. Patient natural history is unfavorable, 64% die during childhood without surgical treatment and 50% in the neonatal period^[4]^.

It consists of a 90° ventricle axis rotation in relation to normal position, meaning ventricles are positioned superoinferiorly instead of anteroposteriorly^[2]^. Diagnosis can be challenging since atrioventricular and ventriculoarterial relationship can present different alterations. This patient presented a concordant atrioventricular connection and a discordant ventriculoarterial connection.

Ventricular axis rotation itself presents no clinical impact; however, there are no reported cases of CCH without association with other malformations. In this case, patient presented TGA with CCH. Symptoms vary depending on existing malformations added to the initial abnormality^[3]^.

The origin of the defect leading to ventricular rotation is uncertain. The relationship of Cx43 gene is being investigated, which deletion would result in delay of cardiac dextroposition, causing ventricle craniocaudal positioning^[3]^.

Diagnosis and surgical treatment become challenging due to its complexity and rarity^[4]^.

A review study evaluating five cases in the prenatal period suggests that detailed evaluation of fetal CCH can result in correct anatomical and pathophysiological diagnoses^[5]^.

Another study shows eight patients with a median gestational age of 27 weeks at diagnosis, born alive, seven with *situs solitus,* and one with *situs ambiguous.* In all cases, ventricular-arterial discordance and additional cardiac anomalies were detected. In 50% of the cases, atrioventricular discordance was detected as well^[6]^.

Levocardia is an extremely rare condition, characterized by a left-sided cardiac apex with abdominal *situs inversus* and usually associated with severe forms of congenital heart disease with poor prognosis^[7]^.

Surgical treatment of patients with CCH consists of repairing associated malformation when possible and or palliative surgery^[8]^. This patient was admitted to our service with a late diagnosis of simple TGA, suggesting an atrial switch operation. Even though there was an additional diagnosis of CCH, Senning operation was chosen. During the surgical procedure, it was necessary to use the *in situ* pedicle autologous pericardium to expand the tunnel between the pulmonary veins and the right atrium, characterizing a modified Senning operation.

### Operation

The operation was performed by median sternotomy with total thymus preservation.

Heparinization at 4 mg/kg. Bicaval and aortic cannulation. Hypothermia at 25° C with 147 minutes of cardiopulmonary bypass and nine minutes of total circulatory arrest.

Opening of right atrium according to the Senning technique and resection of the interatrial septum. Opening of left atrium near the entrance of the right pulmonary veins. In total circulatory arrest, pulmonary veins were isolated with bovine pericardium sutures using 6-0 Prolene®. Coronary sinus roof opening. The lateral portion of the right atrium was sutured below the tricuspid valve at the edge of the interatrial septum, in such a way as to allow the blood flow from the vena cava and coronary sinus to drain into the mitral valve, thus constituting the so-called vena cava tunnel. Finally, the borders of the right pulmonary veins were anastomosed to the *in situ* autologous pericardium used as part of the constituent tunnel to the medial portion of the right atrial wall, which was anastomosed anteriorly to the pericardial sac, thus, allowing the construction of a wide tunnel, without blood flow restrictions from pulmonary veins to the right ventricle. A tricuspid valve repair was performed, showing dysplasia, with anchoring of the septal to the anterior leaflet with separate stitches of 6-0 Prolene®.

**Fig. 5:**
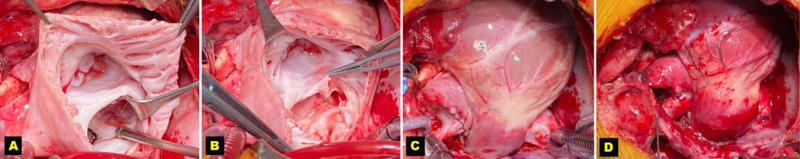
A) Relationship between tricuspid and mitral valves after right atrium opening and resection of the fossa ovalis’ lamina. B) Bovine pericardium patch isolating the pulmonary veins. The forceps pulls the coronary sinus wall after roof sectioning. C) Anastomosis of right atrium in the pedicled pericardial sac. D) Final view of the operation post cardiopulmonary bypass.

Figure 5 (A-D) shows images of the surgical procedure.

In the immediate postoperative period, the patient had an extubation failure secondary to sepsis with a pulmonary focus, also attributed to tricuspid valve insufficiency. On the 12^th^ postoperative day, the patient was extubated. And the patient was discharged one month after surgical procedure in use of furosemide and levothyroxine.

## Abbreviations, Acronyms & Symbols

ASD: Atrial septal defect
CCH: Crisscross heart
TGA: Transposition of the great arteries
